# Exosomal MALAT1 Derived from High Glucose-Treated Macrophages Up-Regulates Resistin Expression via miR-150-5p Downregulation

**DOI:** 10.3390/ijms23031095

**Published:** 2022-01-20

**Authors:** Kou-Gi Shyu, Bao-Wei Wang, Wei-Jen Fang, Chun-Ming Pan, Chiu-Mei Lin

**Affiliations:** 1 Division of Cardiology, Shin Kong Wu Ho-Su Memorial Hospital, Taipei 111, Taiwan; 2 Department of Medical Research, Shin Kong Wu Ho-Su Memorial Hospital, Taipei 111, Taiwan; baowei@ms22.hinet.net (B.-W.W.); t013215@ms.skh.org.tw (W.-J.F.); A009103@ms.skh.org.tw (C.-M.P.); 3 Department of Emergency Medicine, Shin Kong Wu Ho-Su Memorial Hospital, Taipei 111, Taiwan; mei882153@gmail.com; 4 School of Medicine, Fu-Jen Catholic University, New Taipei City 242, Taiwan

**Keywords:** high glucose, macrophage, MALAT1, exosome, miR-1505p, resistin

## Abstract

Metastasis-associated lung adenocarcinoma transcript 1 (MALAT1) plays a crucial role in the pathophysiological process associated with diabetes-related complications. The effect of high glucose levels on macrophage-derived exosomal MALAT1 is unknown. Therefore, we investigated the molecular regulatory mechanisms controlling exosomal MALAT1 in macrophages under high glucose treatment and the therapeutic target of macrophage-derived exosomal MALAT1 using a balloon injury model of vascular disease in diabetic rats. High glucose (25 mM) significantly increased MALAT1 expression in macrophage-derived exosomes. MALAT1 suppressed miR-150-5p expression in macrophage-derived exosomes under high-glucose conditions. Silencing MALAT1 using MALAT1 siRNA significantly reversed miR-150-5p expression induced by macrophage-derived exosomes. Macrophage-derived exosomes under high-glucose treatment significantly increased resistin expression in macrophages. Silencing MALAT1 and overexpression of miR-150-5p significantly decreased resistin expression induced by macrophage-derived exosomes. Overexpression of miR-150-5p significantly decreased resistin luciferase activity induced by macrophage-derived exosomes. Macrophage-derived exosome significantly decreased glucose uptake in macrophages and silencing MALAT1, resistin or overexpression of miR-150-5p significantly reversed glucose uptake. Balloon injury to the carotid artery significantly increased MALAT1 and resistin expression and significantly decreased miR-150-5p expression in arterial tissue. Silencing MALAT1 significantly reversed miR-150-5p expression in arterial tissue after balloon injury. Silencing MALAT1 or overexpression of miR-150-5p significantly reduced resistin expression after balloon injury. In conclusion, high glucose up-regulates MALAT1 to suppress miR-150-5p expression and counteracts the inhibitory effect of miR-150-5p on resistin expression in macrophages to promote vascular disease. Macrophage-derived exosomes containing MALAT1 may serve as a novel cell-free approach for the treatment of vascular disease in diabetes mellitus.

## 1. Introduction

Atherosclerosis-related cardiovascular disease is a major cause of mortality worldwide. The prevalence of diabetes mellitus (DM) is rapidly increasing across the globe, and DM is associated with atherosclerotic cardiovascular disease and heart failure, which may increase the mortality and hospitalization of diabetic patients. It is also known that macrophages play a crucial role in atherosclerosis [1,2].

Long noncoding RNAs (lncRNAs) are non-protein-coding RNAs characterized by at least 200 bp in length with highly conserved sequences [3]. LncRNAs have been shown to regulate the functions of endothelial cells, smooth muscle cells, macrophages, vascular inflammation, and metabolism, suggesting that lncRNAs may influence the progression of atherosclerosis [4]. Metastasis-associated lung adenocarcinoma transcript 1 (MALAT1) is a conserved lncRNA whose expression correlates with many human diseases. MALAT1 has been shown to play an important role in the pathophysiology of diabetes-related complications [5]. MALAT1 has also been shown to regulate hyperglycemia-induced inflammatory processes in endothelial cells [6], and inhibition of MALAT1 down-regulates resistin to reduce insulin resistance by exercise [7]. MALAT1 was significantly up-regulated in the retinas of diabetic mice in a high-glucose-treated retinal endothelial cell line, and in the aqueous humor samples and fibrovascular membranes of diabetic patients [8].

Previous studies have reported that miR-150-5p is the target of MALAT1 in other cell types. It is not known whether miR-150-5p is the target of MALAT1 in macrophage. Resistin, an adipokine, has been reported to be secreted from macrophages and plays an important role in atherosclerosis. High serum resistin level was found in diabetic patients and was correlated with cardiovascular risk. The effect of macrophage-derived exosomal MALAT1 on resistin expression is not clear. The link between miR-150-5p and resistin has not yet been reported. Exosomes are extracellular vesicles of endosomal origin that play a crucial role in intercellular functions [9]. Macrophage-derived exosomes have been reported to impair beta cell insulin secretion [10]. However, the effect of hyperglycemia on the regulation of macrophage-derived exosomal MALAT1 and its possible regulatory mechanism in hyperglycemia have not been previously characterized. Since DM increases the risk of atherosclerosis onset and progression, the possible impact on the field of therapies for diabetes-mediated atherosclerotic patients needs to be studied. We hypothesized that there is a link between MALAT1, miR-150-5p, resistin, and atherosclerosis in high glucose status. In this study, we investigated the molecular regulatory mechanisms associated with exosomal MALAT1 in macrophages under high glucose treatment and the therapeutic target of macrophage-derived exosomes using a balloon injury model of vascular disease in diabetic rats.

## 2. Results

### 2.1. High Glucose Induced MALAT1 Expression in Macrophage-Derived Exosomes

**Figure 1:**
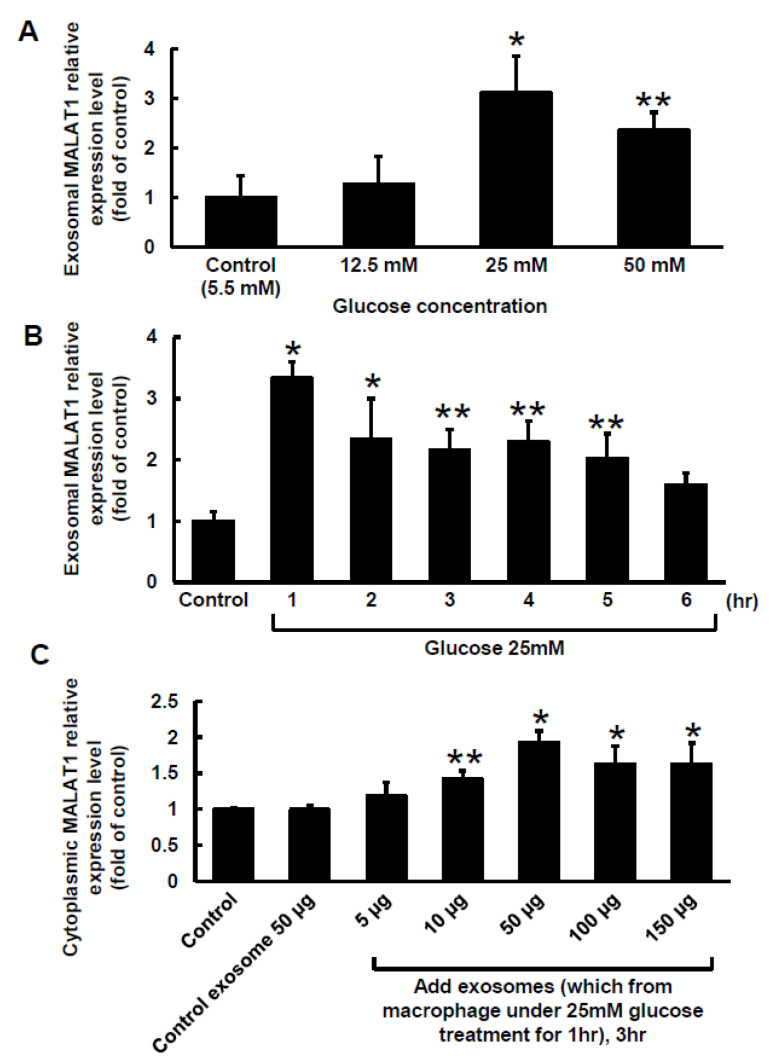
Effect of high glucose on exosomal MALAT1 expression in cultured macrophages. (**A**), Treatment with different glucose concentrations for 1 h. (**B**), Treatment with a glucose concentration of 25 mM for different periods of time. (**C**), Effects of exogenously administering different levels of macrophage-derived exosomes for 3 h on cytosolic MALAT1 expression. The macrophage-derived exosomes were extracted from macrophages under 25 mM glucose treatment for 1 h. * *p* < 0.01 vs. control. ** *p* < 0.05 vs. control. *n* = 4 per group.

Different concentrations of glucose were added to the culture medium for 1 h to explore the effect of high glucose on MALAT1 expression in macrophage-derived exosomes. As shown in Figure 1A, macrophage-derived exosomal MALAT1 significantly increased from 12.5 mM to 50 mM of glucose concentration, and 25 mM high glucose had the maximal effect. Therefore, 25 mM high glucose was used for subsequent experiments. Three concentrations of mannitol were added to exclude the osmotic effect of high glucose on MALAT1 expression. The mannitol did not affect MALAT1 expression (data not shown). As shown in Figure 1B, 25 mM high glucose produced the greatest increase in macrophage-derived exosomal MALAT1 expression at 1 h, but its expression then gradually decreased and reached a level that was similar to the control after 6 h. The exogenous addition of exosomes derived from macrophages under the 25 mM glucose treatment significantly increased cytosolic MALAT1 expression in a dose-dependent manner compared to the control or exosomes derived from control macrophages (Figure 1C). The exogenous addition of exosomes at the 50-µg level produced the largest increase in cytosolic MALAT1. Therefore, 50 µg of exosomes derived from macrophages treated with 25 mM glucose were used for the following experiments. The exosomes derived from macrophages under the 25 mM glucose treatment did not induce significant cytotoxicity when compared to the control (Supplemental Figure S1). The particle size of exosome extracted from macrophages is about 50–150 nm in size.

### 2.2. Macrophage-Derived Exosomes Decreased miR-150-5p and Increased Resistin Expressions in Macrophages

**Figure 2:**
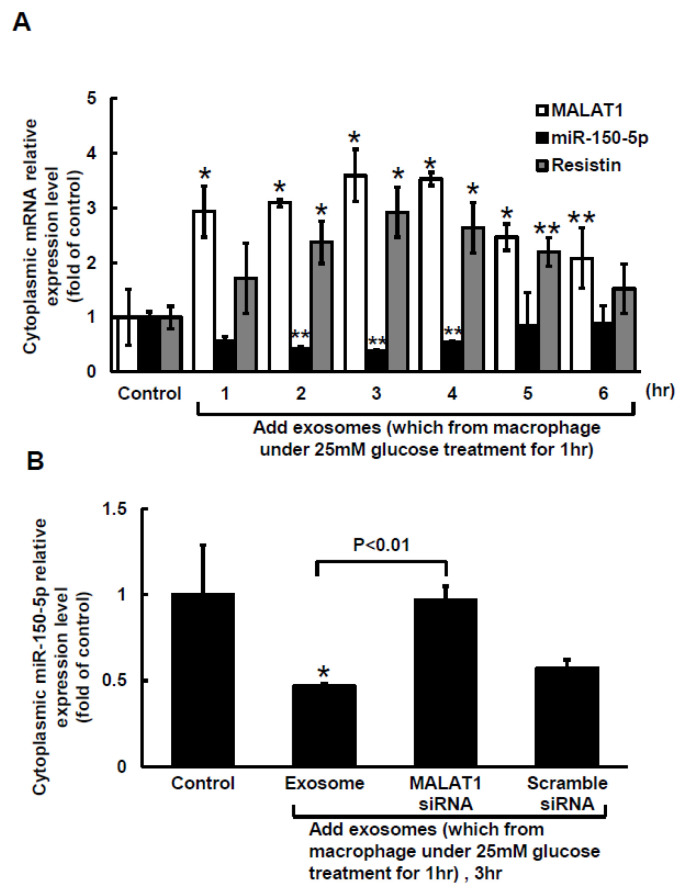
The decrease in miR-150-5p expression in cultured macrophages is mediated by macrophage-derived exosomes. (**A**), exogenous administration of macrophage-derived exosomes for different periods of time. The macrophage-derived exosomes were extracted from macrophages under 25 mM glucose treatment for 1 h. (**B**), Quantitative real-time PCR of cytoplasmic miR-150-5p mRNA levels. Scramble siRNA was the control siRNA. The macrophage-derived exosomes were extracted from macrophages under 25 mM glucose treatment for 1 h. * *p* < 0.01 vs. control. ** *p* < 0.05 vs. control. *n* = 4 per group.

**Figure 3:**
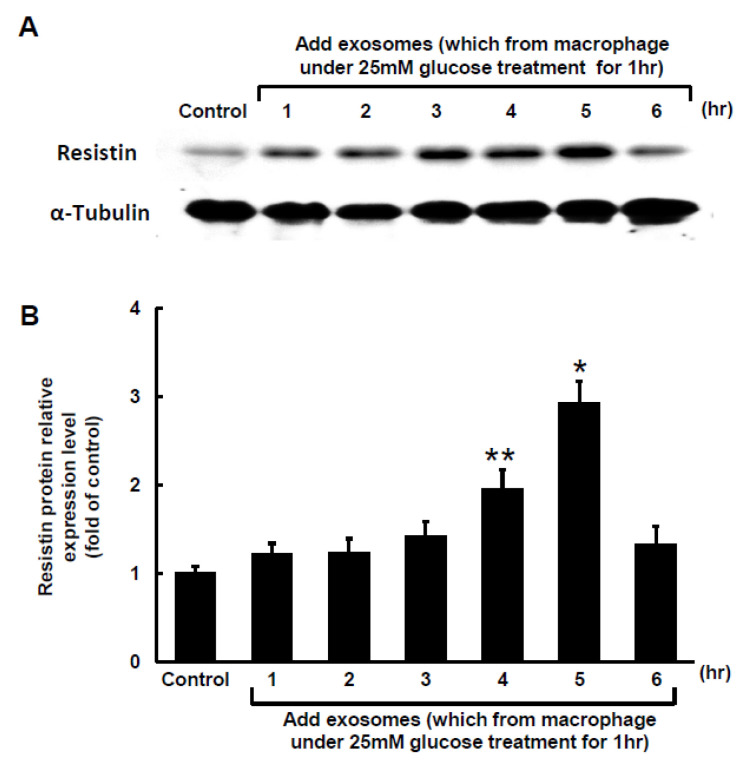

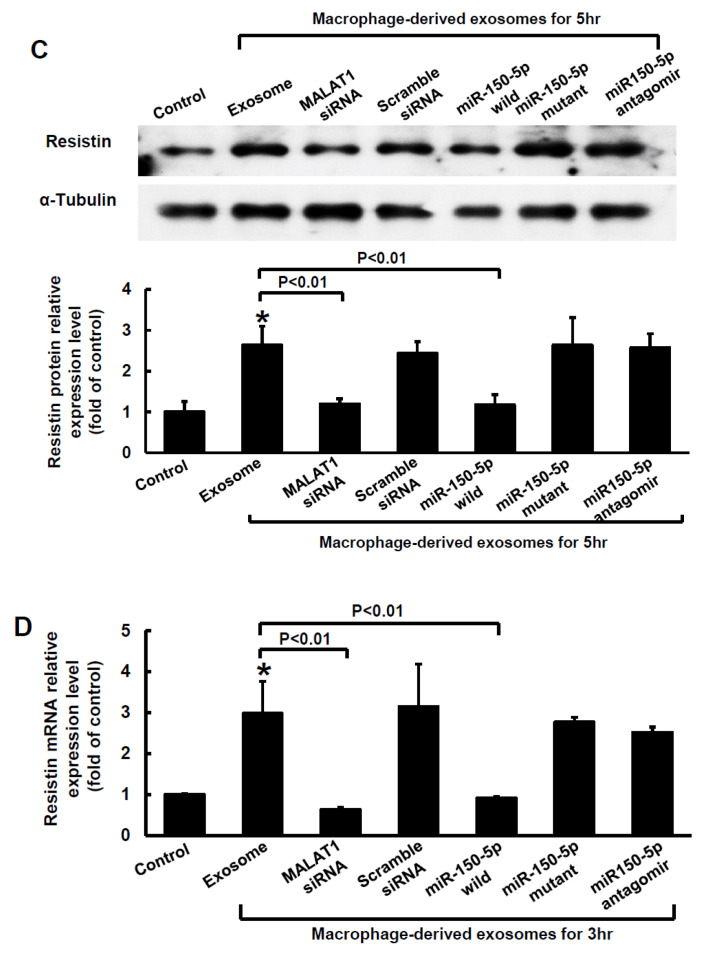
Effect of macrophage-derived exosomes on resistin expression in macrophages. (**A**), Representative Western blots for resistin and α-tubulin protein levels in macrophages subjected to basal glucose concentration (5.5 mM) stimulation for different periods of time. The macrophage-derived exosomes were extracted from macrophages under 25 mM glucose treatment for 1 h. (**B**), Quantitative analysis of resistin protein levels. The macrophage-derived exosomes were extracted from macrophages under 25 mM glucose treatment for 1 h. The values for stimulated macrophages have been normalized to the control cell values. (**C**), Upper panel, representative Western blots for resistin and α-tubulin protein levels in macrophages subjected to basal glucose concentration (5.5 mM) stimulation for different treatments. The macrophage-derived exosomes were extracted from macrophages under 25 mM glucose treatment for 1 h. Lower panel, quantitative analysis of resistin protein levels. The values for the stimulated macrophages have been normalized to the control cell values. (**D**), Quantitative real-time PCR analysis of resistin mRNA levels. The values for the treated macrophages are expressed as a ratio of the normalized values for resistin mRNA in the control cells. MALAT1 siRNA and the overexpression of miR-150-5p significantly reduced resistin expression induced by macrophage-derived exosomes from the basal glucose concentration (5.5 mM) treatment. Scramble siRNA was the control siRNA. The macrophage-derived exosomes were extracted from macrophages under 25 mM glucose treatment for 1 h. * *p* < 0.01 vs. control. ** *p* < 0.05 vs. control. *n* = 4 per group.

The Clustal W method in MegAlign software (DNASTAR, Madison, WI, USA) was used to search for the MALAT1 target microRNA (miR) and discovered that a mouse MALAT1 matching site for miR-150-5p was located between 5243 bp and 5265 bp on the mouse MALAT1 gene loci. The 50-µg macrophage-derived exosomes under 25 mM glucose treatment significantly increased cytoplasmic MALAT1 mRNA levels in macrophages, but the miR-150-5p cytoplasmic mRNA levels significantly decreased after treatment with macrophage-derived exosomes without glucose. Cytoplasmic resistin mRNA levels showed a pattern similar to that of MALAT1 under macrophage-derived exosome treatment (Figure 2A). Silencing MALATA1 with MALAT1 siRNA significantly reversed cytoplasmic miR-150-5p expression in macrophages (Figure 2B). The scramble MALAT1 siRNA did not significantly affect cytoplasmic miR-150-5p mRNA expression under macrophage-derived exosome treatment. Exosomes derived from macrophages under 25 mM glucose treatment significantly increased resistin protein expression for 4 to 5 h, but then the resistin levels returned to normal after 6 h (Figure 3A,B). MALAT1 siRNA significantly reduced resistin protein and mRNA expression in macrophages treated with macrophage-derived exosomes (Figure 3C,D). The scramble MALAT1 siRNA did not significantly affect resistin protein and mRNA expression under macrophage-derived exosome treatment. Overexpression of wild-type miR-150-5p significantly reduced resistin protein and mRNA expression induced by macrophage-derived exosomes, while overexpression of the mutant miR-150-5p or the antagomir of miR150-5p did not reduce resistin protein and mRNA expression induced by macrophage-derived exosomes. These results indicated that macrophage-derived exosomes under high glucose treatment increased MALAT1 expression but decreased miR150-5p expression to enhance resistin expression in macrophages.

### 2.3. MiR-150-5p Is the Target of MALAT1 and Resistin Is the Target of miR-150-5 in Macrophage

**Figure 4:**
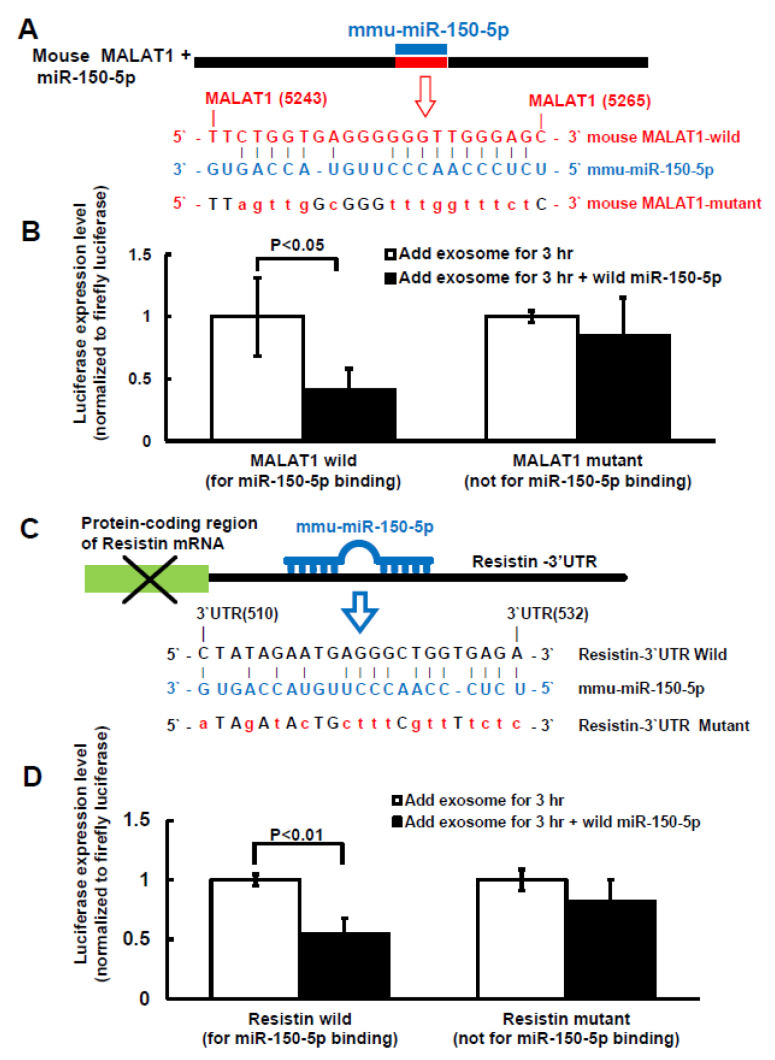
Effect of miR-150-5p on MALAT1 and resistin luciferase activity in macrophages treated with macrophage-derived exosomes. (**A**), Sequence of the mouse MALAT1 3′UTR target site for miR-150-5p binding (nucleotides 5243 to 5265 bp). (**B**), MALAT1 3′UTR luciferase activity under macrophage-derived exosome treatment with wild type or mutant MALAT1. (**C**), Sequence of the mouse resistin 3′UTR target site for miR-150-5p binding, which is located on the resistin 3′UTR (nucleotides 510 to 532 bp). (**D**), Resistin 3′UTR luciferase activity under macrophage-derived exosome treatment with wild type or mutant resistin.

To investigate whether miR-150-5p is the target gene of MALAT1 and resistin is the target gene of miR-150-5p in macrophage under high glucose treatment, MALAT1 and resistin luciferase activity assay was performed. Figure 4A shows the sequence of the MALAT1 3′UTR target site for miR-150-5p binding (nucleotides 5243–5265). MiR-150-5p overexpression significantly decreased MALAT1 luciferase activity in macrophages under 25 mM high glucose treatment when miR-150-5p was bound to the normal MALAT1 3′UTR (Figure 4B). Overexpression of mutant miR-150-5p did not significantly affect MALAT1 luciferase activity, indicating that the MALAT1 target is miR150-5p. We discovered that the resistin 3′UTR (nucleotides 510–532) had a binding site for miR-150-5p, as indicated in Figure 4C, using the Clustal W method in MegAign software (DANSTAR, Madison, WI, USA). MiR-150-5p overexpression significantly decreased resistin luciferase activity in macrophages under 25 mM high glucose treatment when miR-150-5p was bound to the normal resistin 3′UTR (Figure 4D). Overexpression of mutant miR-150-5p did not significantly affect resistin luciferase activity, indicating that resistin is the miR150-5p target gene.

### 2.4. Macrophage-Derived Exosomes Reduced Glucose Uptake in Macrophages

Exogenous addition of macrophage-derived exosomes under the 25 mM high glucose treatment significantly reduced glucose uptake in macrophages. Furthermore, silencing MALAT1 using MALAT1 siRNA before the macrophage-derived exosomes treatment significantly reversed glucose uptake to levels that were similar to that in the control cells (Supplemental Figure S2). MALAT1 scramble siRNA did not significantly affect glucose uptake compared to the addition of macrophage-derived exosomes. MiR-150-5p overexpression significantly reversed glucose uptake in macrophages before macrophage-derived exosome treatment. In addition, overexpression of mutant miR-150-5p or the miR-150-50 antagomir before macrophage-derived exosome treatment did not significantly affect glucose uptake.

### 2.5. Balloon Injury Increased MALAT1 to Suppress miR150-5p Expression in Diabetic Rats

**Figure 5:**
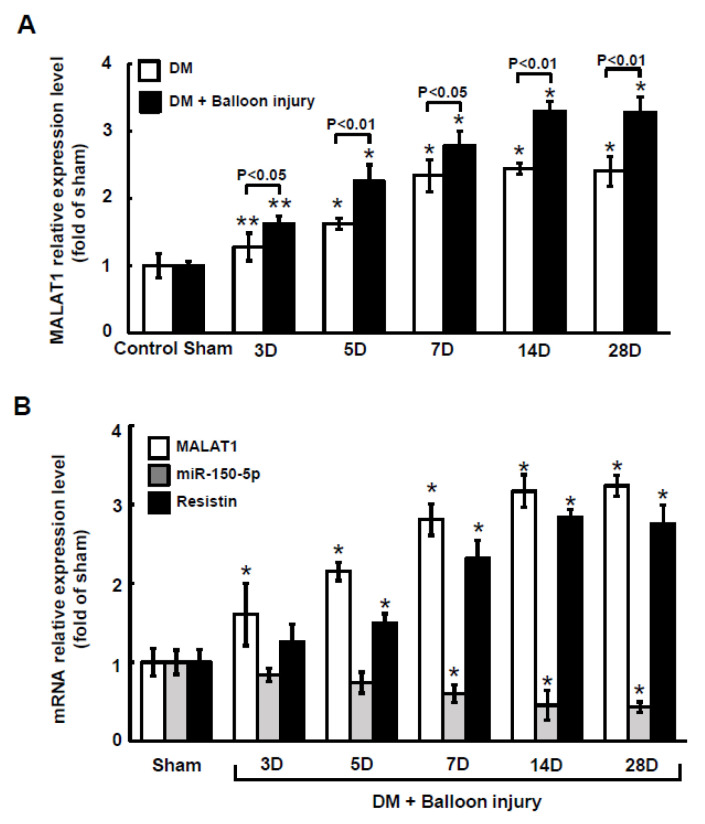
Effect of carotid artery balloon injury on MALAT1 expression in diabetic rats. (**A**), Quantitative real-time PCR analysis of MALAT1 levels in arterial tissue with or without carotid artery balloon injury in diabetic rats after different periods of time. (**B**), Quantitative real-time PCR analysis of MALAT1, miR-150-5p, and resistin mRNA levels in arterial tissue after carotid artery balloon injury for different periods of time. * *p* < 0.01 vs. control. ** *p* < 0.05 vs. control. *n* = 5 per group.

**Figure 6:**
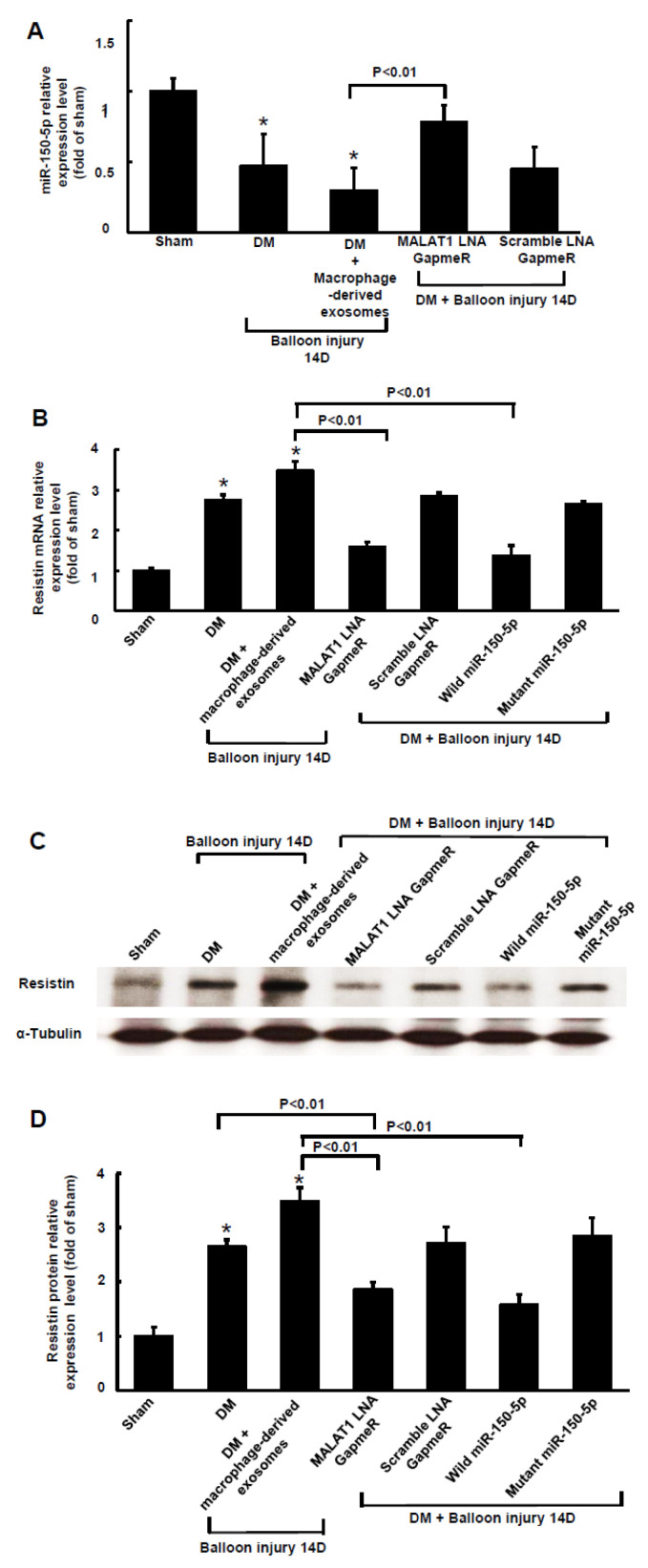
Macrophage-derived exosomal MALAT1-mediates the reduction of miR-150-5p expression after carotid artery balloon injury in diabetic rats. (**A**), Quantitative real-time PCR analysis of miR-150-5p levels in arterial tissue at 14 days after carotid artery balloon injury in diabetic rats. The macrophage-derived exosomes were extracted from macrophages under 25 mM glucose treatment for 1 h. Scramble siRNA was the control siRNA. (**B**), Quantitative real-time PCR of resistin mRNA levels in arterial tissue at 14 days after carotid artery balloon injury in diabetic rats. The macrophage-derived exosome was extracted from macrophages under 25 mM glucose treatment for 1 h. Scramble LNA GapmeR is a control siRNA. (**C**), Representative Western blots for resistin and α-tubulin protein levels in arterial tissues at 14 days after carotid artery balloon injury in diabetic rats. (**D**), Quantitative analysis of resistin protein levels. Scramble LNA GapmeR was the control siRNA. * *p* < 0.01 vs. control. *n* = 5 per group.

MALAT1 expression in arterial tissue significantly increased from 3 days to 28 days in diabetic rats compared to that in wild-type rats (control). MALAT1 expression in arterial tissue also significantly increased from 3 days to 28 days in diabetic rats after balloon injury of the carotid artery compared to diabetic rats without balloon injury (Figure 5A). As shown in Figure 5B, MALAT1 mRNA expression significantly increased and miR-150-5p significantly decreased from 3 days to 28 days after balloon injury of the carotid artery in diabetic rats. The expression pattern for resistin mRNA after balloon injury of the carotid artery was similar to MALAT1 mRNA expression after balloon injury of the carotid artery. Treatment with macrophage-derived exosomes further reduced miR-150-5p expression after balloon injury at 14 days, and silencing MALAT1 by MALAT1 siRNA significantly reversed miR-150-5p expression after balloon injury at 14 days (Figure 6A). Scramble MALAT1 siRNA did not significantly affect miR-150-5p expression compared to diabetic rats with balloon injury. Treatment with macrophage-derived exosomes further increased resistin mRNA and protein expression at 14 days after balloon injury, and silencing MALAT1 by MALAT1 LNA GapmeR and miR-150-5p overexpression significantly decreased resistin expression at 14 days after balloon injury of the carotid artery compared to the macrophage-derived exosomes treatment group with balloon injury (Figure 6B–D). Overexpression of the scramble LNA GapmeR or mutant miR-150-50 did not significantly affect resistin mRNA and protein expression compared to the macrophage-derived exosomes treatment group with balloon injury.

### 2.6. Macrophages-Derived Exosomes Increased Resistin Protein Expression in the Arterial Tissue of Diabetic Rats after Carotid Artery Balloon Injury

**Figure 7:**
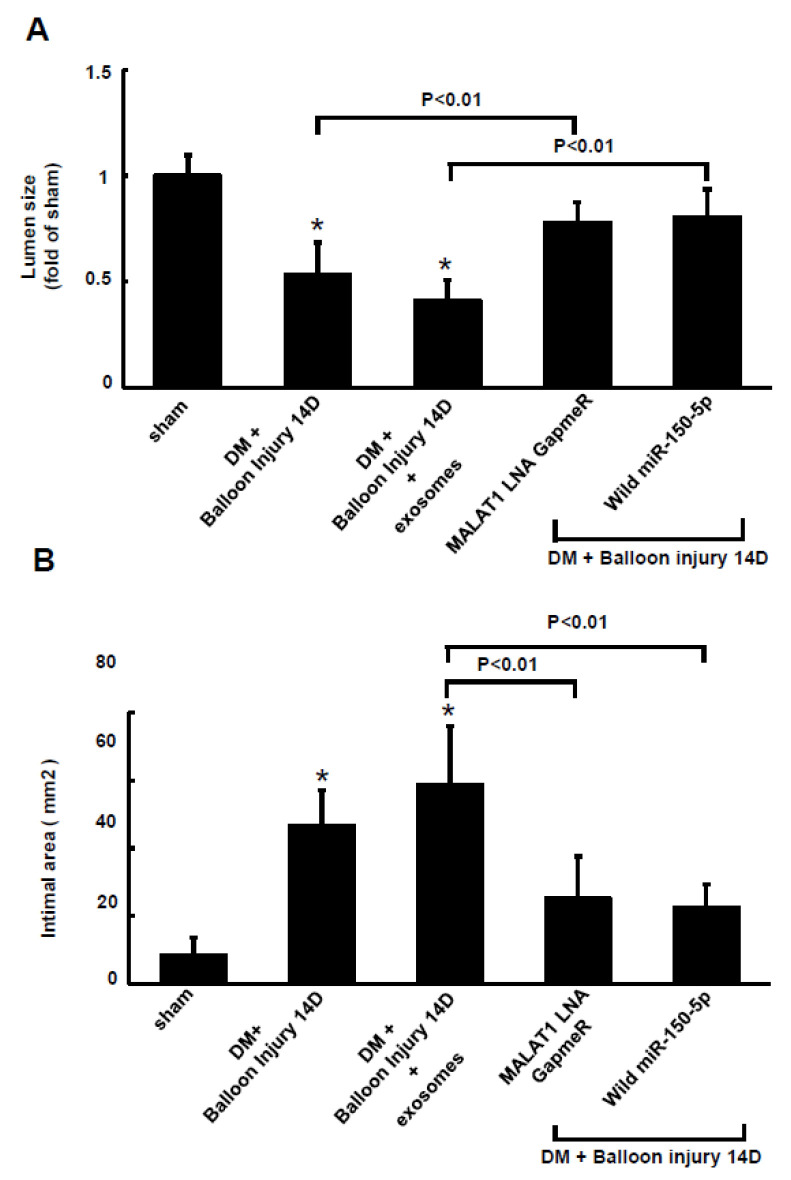
Lumen size and intimal area of the carotid artery after balloon injury in diabetic rats. Lumen size (**A**) and intimal area (**B**) of the carotid artery at 14 days after balloon injury. The macrophage-derived exosomes were extracted from macrophages under 25 mM glucose treatment for 1 h. * *p* < 0.01 vs. control. *n* = 5 per group.

The resistin fluorescent signals increased in the arterial tissue at 14 days after balloon injury of the carotid artery in diabetic rats. Treatment with macrophage-derived exosomes also increased the resistin fluorescent signals (Supplemental Figure S3). Silencing MALAT1 by MALAT1 LNA GapmeR and miR-150-5p overexpression decreased the resistin fluorescent signals after balloon injury at 14 days compared to the macrophage-derived exosomes treatment group with balloon injury. Balloon injury significantly decreased lumen size and increased the intimal area of the carotid artery, whereas treatment with MALAT1 LNA GapmeR to silence MALAT1 and overexpression of wild-type miR-150-5 significantly increased lumen size and decreased the intimal area compared to the macrophage-derived exosomes treatment group (Figure 7A,B).

## 3. Discussion

Patients with DM have a higher risk of atherosclerotic cardiovascular disease and its associated morbidity and mortality [11]. Macrophages play a key role in the pathogenesis of vascular disease [1]. LncRNAs participate in the progression of vascular disease [4] and MALAT1 plays a crucial role in the pathophysiological process associated with diabetes-related complications [5]. High glucose levels can increase MALAT1 expression in endothelial cells [6] and MALAT1 up-regulates resistin to reduce insulin resistance during exercise [7]. Although Han et al. reported that hyperglycemia up-regulated MALAT1 expression in macrophages [12], the effect of hyperglycemia on the regulation of macrophage-derived exosomal MALAT1 expression is currently unknown. In the present study, we demonstrated that high glucose significantly increased macrophage-derived exosomal MALAT1 expression and that macrophage-derived exosomal MALAT1 regulated miR-150-5p and resistin expression.

Previous studies have shown that MALAT1 acts as a sponge for miR150-5p in chondrocytes, human umbilical vein endothelial cells, H9c2 cells, and human pulmonary microvascular endothelial cells [13,14,15,16]. In this study, we demonstrated that MALAT1 decreased miR-150-5p expression in macrophages under high glucose stimulation. We also found that the MALAT1 promoter had a miR-150-5p binding site. The macrophage-derived exosomes treated with 25 mM glucose for 1 h contained MALAT1. Furthermore, the addition of macrophage-derived exosomes reduced miR-150-5p expression, and the silencing of MALAT1 by MALAT1 siRNA reversed miR-150-5p expression. 

Resistin, known as an adipokine, contributes to insulin resistance in rodents [17] and has been shown to have pro-atherosclerotic effects by inducing vascular inflammation, lipid accumulation, and plaque vulnerability [18]. Resistin has been reported to be secreted from macrophages in atheroma and promotes vascular change [19]. Our study showed that macrophage-derived exosomes containing MALAT1 could bind to miR-150-50 to up-regulate resistin protein expression, and silencing MALAT1 significantly reversed the exosome effect. Overexpression of miR-150-5p also significantly reversed the exosome effect. The resistin promoter had a miR-150-5p binding site, and the overexpression of miR-150-5p significantly decreased resistin luciferase activity in macrophages following exosome treatment. The results indicated that resistin is a target gene of miR-150-5p. Recently, Liu et al. reported that inhibition of MALAT1 reduced resistin expression via elevation of miR-382-3p in human umbilical endothelial cells to reduce insulin resistance [7]. Macrophage-derived exosomal MALAT1 also decreased glucose uptake, and silencing MALAT1 or resistin and overexpression of miR 150-5p reversed glucose uptake in macrophages. These results indicate that macrophage-derived exosomal MALAT1 may induce insulin resistance or impair insulin secretion. Recently, Qian et al. reported that macrophage-derived exosomes could impair beta cell insulin secretion in mice [10].

Our in vivo carotid artery balloon injury model demonstrated that macrophage-derived MALAT1 could promote carotid artery plaque formation induced by balloon injury. MALAT1 was induced in the carotid arterial tissue of diabetic rats, and balloon injury further increased MALAT1 expression in these rats. Balloon injury of the carotid artery decreased miR-150-5p expression and increased resistin expression in diabetic rats, but treatment with macrophage-derived exosomes decreased miR-150-5p expression and increased resistin expression during balloon injury of the carotid artery. Silencing MALAT1 reversed miR-150-5p expression and reduced resistin expression in the carotid artery after balloon injury. Balloon injury of the carotid artery also decreased the lumen size and increased the intimal area. In contrast, silencing MALAT1 or overexpression of miR-150-5p increased lumen size and reduced intimal area. The result of our in vivo study may be preliminary because we did not investigate the characteristics of in juried artery with or without exosomes treatment.

However, the effect of MALAT1 on vascular disease remains controversial. Gast et al. and Cremer et al. reported that MALAT1 deficiency promoted plaque formation and plaque inflammation in ApoE-deficient mice [20,21], Chen et al. reported that MALAT1 overexpression attenuated plaque formation by inhibiting oxidized low-density lipoprotein-stimulated dendritic cell maturation [22]. However, some studies have reported that MALAT1 induces vascular disease [12,23,24,25,26]. Han et al. reported that MALAT1 promoted diabetic vascular disease by increasing the pyroptosis of macrophages in rats [12]; Song et al. reported that MALAT1 promoted high glucose-induced pyroptosis in human endothelial cells to induce vascular disease [23]; and Wang et al. reported that MALAT1 was up-regulated in patients with vascular disease and that suppression of MALAT1 protected the endothelium from oxidized low-density lipoprotein-induced inflammation and oxidative stress in endothelial cells [24]. Zhu et al. reported that MALAT1 was overexpressed in blood samples from patients with coronary atherosclerotic heart disease, and knockdown of MALAT1 suppressed the development of vascular disease in mice [25]. Gao et al. reported that exosomal MALAT1 derived from oxidized low-density lipoprotein-treated endothelial cells aggravated vascular disease in mice [26]. Our study demonstrated that diabetic rats increased MALAT1 expression and silencing MALAT1 decreased intimal hyperplasia after balloon injury of the carotid artery. The use of different cell types and in vivo models may explain the discrepancy in the effect that MALAT1 has on the promotion or attenuation of vascular disease.

**Figure 8:**
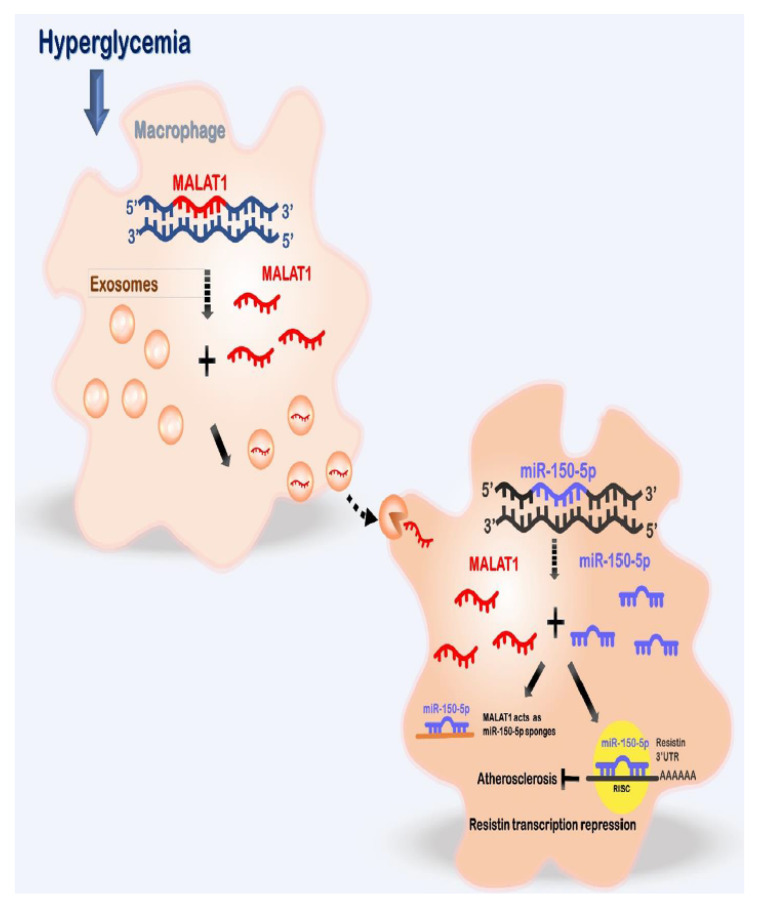
Proposed pathway for enhanced vascular disease mediated by high glucose -induced macrophage-derived exosomes through the up-regulation ofMALAT1, consequent suppression of miR-150-5p expression and up-regulation of resistin expression in macrophages. This pathway illustrates the critical role of macrophage-derived exosomal MALAT1 in vascular disease in diabetes mellitus. Therefore, macrophage-derived exosomal MALAT1 can serve as a valuable therapeutic target for vascular disease therapy in diabetes mellitus.

Macrophage-derived exosomes play a crucial role in vascular disease [27,28]. Wang et al. reported that macrophage-derived exosomes were involved in the pathogenesis of abdominal aortic aneurysms [27]. Exosomes are stable, safe, and effective carriers that contain many crucial proteins and microRNAs that promotes vascular disease. Our study indicates that macrophage-derived exosomes containing MALAT1 may serve as a novel cell-free approach for the treatment of vascular disease caused by diabetes mellitus. The effect of high glucose on MALAT1, miR-150-5p, and resistin in macrophages is summarized in Figure 8. A better understanding of the detailed mechanisms associated with the therapeutic targets of MALAT1 under hyperglycemic conditions will provide new insights into the therapeutic development of vascular disease that is frequently encountered in patients suffering from diabetes mellitus. Macrophages have different phenotypes under different stimulation. M1 macrophages are pro-inflammatory and M2 macrophages are anti-inflammatory [29]. It has been reported that exosome-guided phenotypic switch of MI to M2 macrophages could improve wound healing [30]. In this study, we did not check our macrophages phenotype. Based on our study findings, we speculate that our macrophage phenotype is M1 because it aggravates vascular disease. Glycemic control is critical to diabetes management because uncontrolled diabetes is associated with significant subclinical vascular disease and an increased risk for cardiac events as compared with controlled diabetes [31].

There are several limitations of our study. Firstly, the use of rats as model was examined only briefly after exosome treatment and hyperglycemia induction. Secondly, the carotid artery balloon injury induces intimal hyperplasia and is taken as a surrogate for vascular disease. Although intimal hyperplasia is not exactly vascular disease, it indicates an early sign of vascular disease. The nature of the arterial injury was not examined. Thirdly, our study did not induce hyperlipidemia which is usually associated with vascular disease. However, not all diabetic patients have hyperlipidemia, and vascular disease could be induced by other factors such as inflammation, diabetes, smoking, and high blood pressure. Fourthly, the characterization of macrophage phenotype was not identified. Fifthly, resistin expression in adipose tissue could be affected by MALAT1. The present study did not investigate adipose tissue in carotid artery.

## 4. Materials and Methods

### 4.1. Cell Culture

The macrophages (RAW264.7) were purchased from the Bioresource Collection and Research Center (BCRC, Hsinchu, Taiwan). The RAW264.7 line was derived from *Mus musculu* (mouse). The macrophages were then cultured in Dulbecco’s modified Eagle’s medium (DMEM, Thermo Fisher Scientific, Waltham, MA, USA) supplemented with 10% fetal bovine serum (Gibco, Co., Dublin, Ireland) at 37 °C with 5% CO_2_. The cells were grown to 80–90% confluence in 10 cm culture dishes and sub-cultured at a ratio of 1:3. The exosomes were collected by removing 5 mL of cell-free supernatant from the macrophage culture after treatment with 25 mM glucose.

### 4.2. Hyperglycemic Stress Environment

The cells were seeded in 10 cm culture dishes in cell growth medium with different glucose concentrations from 5.5 mM to 75 mM.

### 4.3. Extraction of Exosomes from Cell Media 

Exosome extraction from the cell culture media was performed using Total Exosome Isolation Reagent (Invitrogen, Thermo Fisher Scientific, Waltham, MA, USA) according to the manufacturer’s instructions. The procedure was performed as previously prescribed [32]. The amounts of exosomes were quantitated using an ExoQuantTM quantification assay kit according to the manufacturer’s instructions (BioVision, Milpitas, CA, USA).

### 4.4. RNA Quality Assessment of the Exosomes

A macrophage-derived exosomal MALAT1 RNA quality assessment was performed using a BiOptic Qsep100 using RNA cartridge kit (BiOptic Inc., Taipei, Taiwan), following the manufacturer’s protocol. An electropherogram and a virtual gel image were generated to visualize the samples and improve interpretation. All samples were analyzed in duplicate.

### 4.5. Reverse Transcription and Real-Time Quantitative Polymerase Chain Reaction

Exosomal MALAT1 RNA extraction was carried out by Total Exosome RNA and Protein Isolation Kit (Invitrogen, Thermo Fisher Scientific, Waltham, MA, USA) according to the manufacturer’s protocol. To quantify the MALAT1-exosome RNA transcripts, 12 μL of a 14 μL RNA eluate was subjected to reverse transcription with random hexamers using a High-Capacity cDNA Reverse Transcription kit (Applied Biosystems, Thermo Fisher Scientific, Waltham, MA, USA). For the quantitative polymerase chain reaction (qPCR), 10% of each cDNA reaction was analyzed using standard SYBR chemistry and cycler conditions, and Fast SYBR^®^ Green Master Mix (Applied Biosystems, Thermo Fisher Scientific, Waltham, MA, USA) was used for further assays. The PCRs were performed on an ABI StepOnePlus cycler with a 96 well block and the following two-step cycler profile: 95 °C for 15 min, 40 × (94 °C for 15 s, 55 °C for 30 s, and 70 °C for 30 s. Relative gene expression levels were analyzed using the formula 2–ΔCT with ΔCT = CT (target gene) − CT (control). The DNA sequences of the individual PCR products were analyzed to verify the purity of the product.

### 4.6. Polymerase Chain Reaction Product Construction and Sequencing 

Cloning was performed using the pGEM^®^-T Easy Vector System (Promega, Madison, WI, USA). Then, ligation was carried out in a final reaction volume of 10 μL consisting of 5 μL of 2X Rapid Ligation Buffer, 1 μL of pGEM^®^-T or pGEM^®^-T Easy Vector (50 ng), 1 μL of T4 DNA ligase, and 3 μL of PCR product. The sample was incubated in a thermocycler for 1 h at 25 °C and then further incubated overnight at 4 °C. The reaction mix was added to 100 µL chemically competent DH5α cells, incubated for 15–30 min on ice, and transformed by heat shock. Liquid LB medium (800 µL) was then added to the transformation solution and the cells were allowed to recover for 45 min at 37 °C. Aliquots of the transformation solution were plated on LB plates containing ampicillin, IPTG, and X-Gal and incubated overnight (16–24 h) at 37 °C. The white colonies were screened for inserts by a colony PCR using the M13 forward primer and the M13 reverse primer. The PCR products were analyzed by sequencing. The sequencing reactions were performed using a BigDye Terminator v3.1 Cycle Sequencing kit (Applied Biosystems, Foster City, CA, USA). Each sequencing reaction was amplified in a 10-μL reaction mixture containing 10–30 ng of amplified DNA. The thermal cycling profile consisted of an initial denaturation step at 95 °C for 10 s, 25 cycles of denaturation at 95 °C for 10 s, annealing at 50 °C for 5 s, and extension at 60 °C for 1 min. The primers used for the direct sequencing reactions were identical to those used in the amplification reactions and the nucleotide sequences for both strands were determined using an ABI 3730 Genetic Analyzer (Applied Biosystems, Foster City, CA, USA) equipped with a long-read sequencing capillary and a POP-7 sequencing polymer.

### 4.7. Western Blot Analysis 

The cells were harvested by scraping and then centrifuged (300× *g*) for 10 min at 4 °C. The pellet was re-suspended and homogenized in lysis buffer (Promega, Madison, WI, USA) and centrifuged at 10,600× *g* for 20 min. A Bio-Rad Protein Assay was used to measure the protein content. Equal amounts of protein (30 μg) were loaded into a 10% SDS-polyacrylamide-mini-gel, followed by electrophoresis. The procedure was performed as previously described [32].

### 4.8. Transfection of MALAT1 Locked Nucleic Acid (LNA) GapmeR 

The cells were transfected at 60% to 75% confluence with 5 nM synthesized small interfering RNAs (siRNA) targeting mouse MALAT1 (Cat. no. 4392420, Thermo Fisher Scientific, Waltham, MA, USA), or 5 nM siRNA targeting mouse resistin (sc-39723, Santa Cruz Biotechnology, Santa Cruz, CA, USA) using Lipofectamine RNAiMax (Thermo Fisher Scientific, Waltham, MA, USA) according to the manufacturer’s protocol. Locked nucleic acid (LNA) GapmeR targeting MALAT1 (50 nM) (Cat. no. 339511 LG00157651-DDA, Qiagen, Valencia, CA, USA) was used in the in vivo study, and scrambled siRNA or LNA GapmeR was the negative control.

### 4.9. Luciferase Activity Assay

A 500 bp mouse resistin-3′UTR DNA fragment (Chromosome 8: 3,655,770-3,658,096; http://www.ensembl.org/index.html, 11 April 2021), and a mouse MALAT1 DNA fragment (Chromosome 19: 5,795,690-5,802,672; http://www.ensembl.org/index.html, 29 March 2021) were generated by artificially synthesizing and cloning them into the pUC57 vector. The clone was digested with SacI and XbaI restriction enzymes and ligated into a pmirNano GLO luciferase plasmid vector. The mouse resistin-3′UTR contains miR-150-5p conserved sites at the resistin-3′UTR (510–532 bp). The mutant was created by mutating the conserved sites, CTATAGAATGAGGGCTGGTGAGA, into ATAGATACTGCTTTCGTTTTCTC and was then constructed using the same method. Mouse MALAT1 contains miR-150-5p conserved sites (from 5243 to 5265 bp in MALAT1). In the mutant, the conserved sites TTCTGGTGAGGGGGGTTGGGAGC were mutated into TTAGTTGGCGGGTTTGGTTTCTC and constructed using the same method. All cloned plasmids were confirmed by DNA sequencing (Seeing Bioscience Co., Ltd., Taipei, Taiwan). The constructed plasmids (2 μg) were transfected with ViaFect™ Transfection reagent (Promega, Madison, WI, USA) according to the manufacturer’s protocol. The luciferase activity was measured as previously described [32].

### 4.10. Construction of the mmu-miR-150-5p Expression Vector

A 165 bp mmu-miR-150-5p precursor (mouse Chromosome 7:45121757-45121821; http://www.ensembl.org/index.html, 20 October 2020) construction was generated using the following method. A 165 bp DNA fragment was generated through artificial synthesis and cloning into a pUC57 vector. The clone was digested with EcoRI and BamHI restriction enzymes and ligated into the pmR-ZsGreen1 plasmid vector (co-expression miR-150-5p and green fluorescent protein) (Clontech Laboratories, Mountain View, CA, USA). The 165-bp synthesis antagomiR-miR-150-5p and the mutant miR-150-5p precursor constructs were generated in the pmR-ZsGreen1 plasmid vector using the same method used for the 165 bp DNA fragment. All cloned plasmids were confirmed by DNA sequencing (Seeing Bioscience Co., Ltd., Taipei, Taiwan). The mutant-miR-150-5p precursor sequence was mutated from TCTCCCAACCCTTGTACCAGTG in the miR-150-5p precursor construct to GAGAAACAAAAGTGGAACCGTT, and the antagomiR-miR-150-5p precursor sequence was mutated from TCTCCCAACCCTTGTACCAGTG in the miR-150-5p precursor construct to CACTGGTACAAGGGTTGGGAGA. The constructed plasmids (2 μg) were transfected with ViaFect™ Transfection reagent (Promega, Madison, WI, USA) in accordance with the manufacturer’s protocol. Briefly, the transfection reagent and plasmid DNA mixture was incubated for 20 min at room temperature. The mixture was then added to the culture cell medium and incubated at 37 °C for 24 h.

### 4.11. Balloon Injury of Carotid Artery in Diabetic Rat and Macrophage-Derived MALAT1-Contained Exosomes Delivery

Male Wistar rats (230–260 g), aged 16 weeks, were injected with a single intraperitoneal injection of streptozotocin at 90 mg/kg (STZ, Sigma, St. Louis, MO, USA) to become diabetic [33]. Diabetes was confirmed by the presence of hyperglycemia (19 mmol/L) for at least 1 week. The balloon injury of the carotid artery was performed 1 week after STZ injection. Adult Wistar rats were anaesthetized with isoflurane (2%) and subjected to balloon catheter injury of the right carotid artery. Briefly, a 2F Forgarty balloon catheter (Biosensors International) was inserted through the right external carotid artery, inflated, and passed three times along the length of the isolated segment (1.5–2 cm in length); then the catheter was removed. MALAT1-contained exosomes (70 µg/150 uL), MALAT1 LNA GapmeR at 50 nM, or 10µg of plasmid DNA was injected to the segment, and electric pulses using CUY21- EDIT Square Wave Electroporator (Nepa Gene) were administered with five pulses and five opposite polarity pulses at 250 V/cm, 50 ms duration, 75 ms interval using Parallel fixed platinum electrode (CUY610P2–1, 1 mm tip, 2 mm gap). The injected MALAT1-containing exosomes or MALAT1 LNA GapmeR was incubated for 10 min. After incubation, unbound MALAT1-containing exosomes or MALAT1 LNA GapmeR was aspirated. The carotid artery was then tied off, and the wound was closed. The rats were sacrificed at 3 to 28 days after balloon injury and the carotids were perfusion fixed at constant physiologic pressure with 4% paraformaldehyde. The vessels were excised and embedded in paraffin blocks, and two cross-sections were cut at positions 1 and 2 cm upstream of the carotid bifurcation. The carotid artery was harvested and fixed in 10% formaldehyde and sliced into 5 μm paraffin sections. Then immunofluorescence staining was performed as previously described [34]. Intimal, medial, and adventitial cross-sectional areas were measured by image software (Nikon NIS-Elements, Tokyo, Japan). Animal experiments were approved by the Institutional Animal Care and Use Committee of Shin Kong Wu Ho-Su Memorial Hospital (Approval number: 109MOST0001) and carried out in accordance with the Guide for the Care and Use of Laboratory Animals.

### 4.12. Statistical Analysis

All results were expressed as mean ± SEM. Statistical significance was evaluated with analysis of variance (GraphPad Software Inc., San Diego, CA, USA). To compare multiple groups to a single control group, Dunnett’s test was used. For pairwise comparisons between multiple groups after the ANOVA, Tukey–Kramer comparison was used. A value of *p* < 0.05 was considered to denote statistical significance.

## 5. Conclusions

Our study shows that high glucose up-regulates MALAT1 to suppress miR-150-5p expression and counteracts the inhibitory effect of miR-150-5p on resistin expression in macrophages to prevent vascular disease. This suggests that macrophage-derived exosomes containing MALAT1 may serve as a novel cell-free approach for the treatment of vascular disease in diabetes mellitus.

## Data Availability

The data that support the findings of this study are available from the corresponding author upon reasonable request.

## Supplementary Materials

The following supporting information can be downloaded at: https://www.mdpi.com/article/10.3390/ijms23031095/s1.
