# Supplementary material for: Exosomal MALAT1 Derived from High Glucose-Treated Macrophages Up-Regulates Resistin Expression via miR-150-5p Downregulation
Source: Int J Mol Sci. 2022 Jan 20;23(3):1095. doi: 10.3390/ijms23031095 (PMC8834900; doi:10.3390/ijms23031095)
Supplement: Supplementary file 1 [file ijms-23-01095-s001.zip › ijms-1460714-supplementary.pdf]

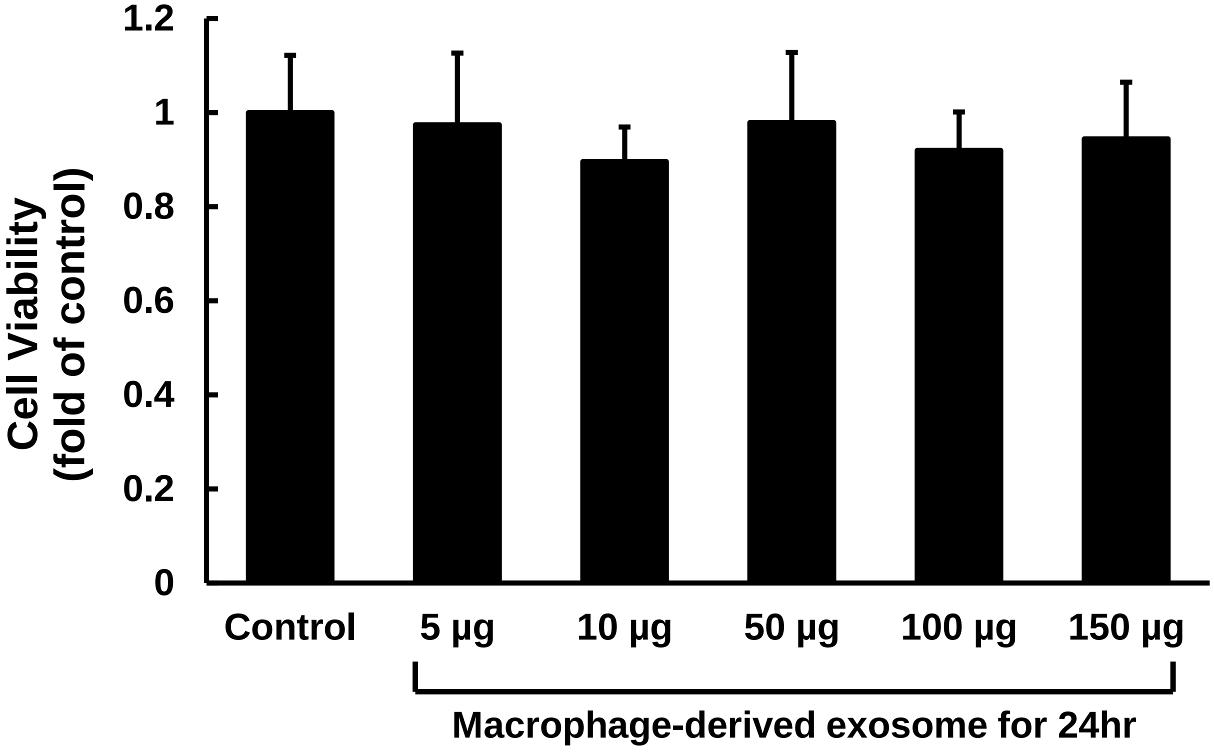

Supplemental Fig.SI

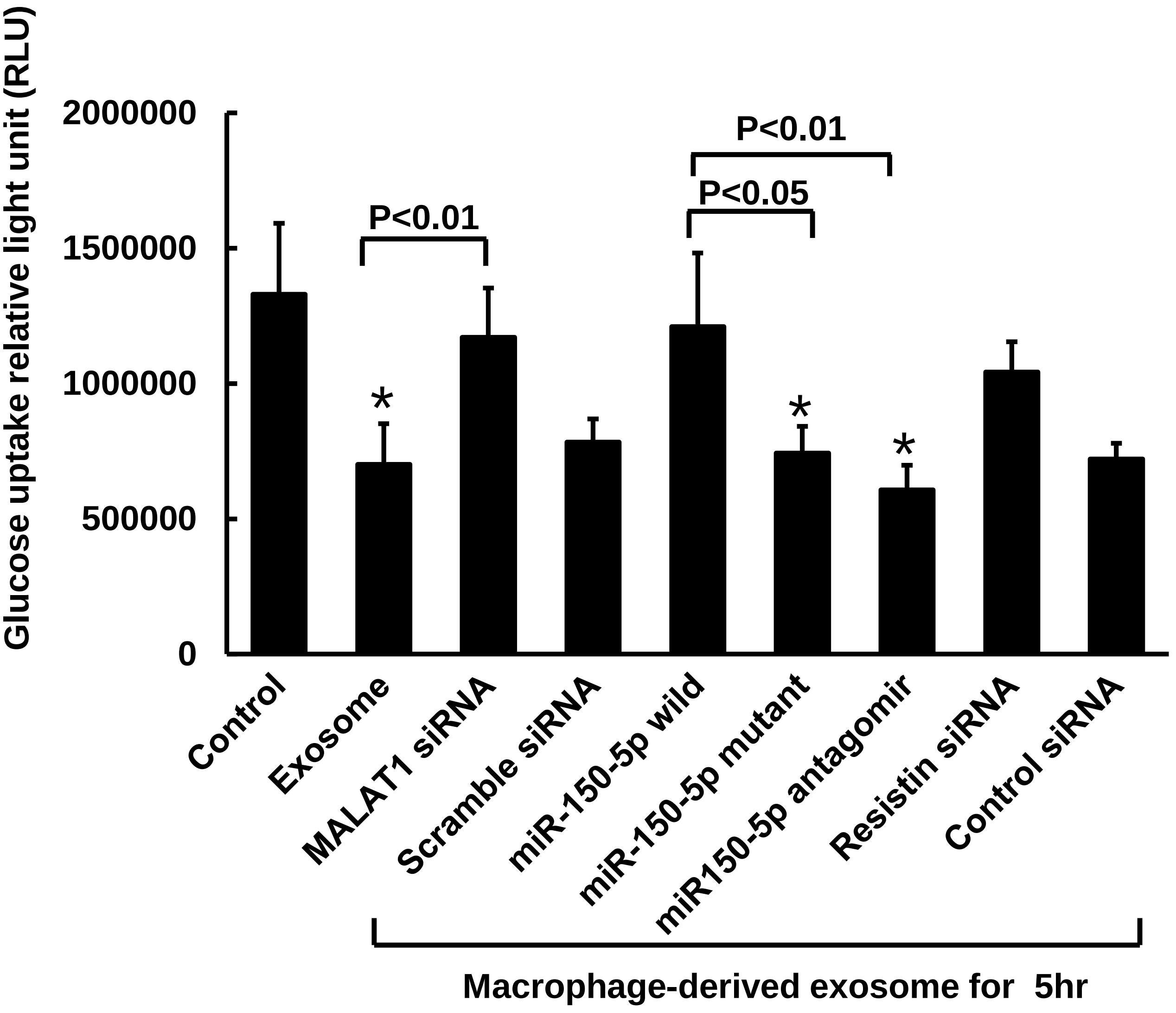

Supplemental Fig.SII

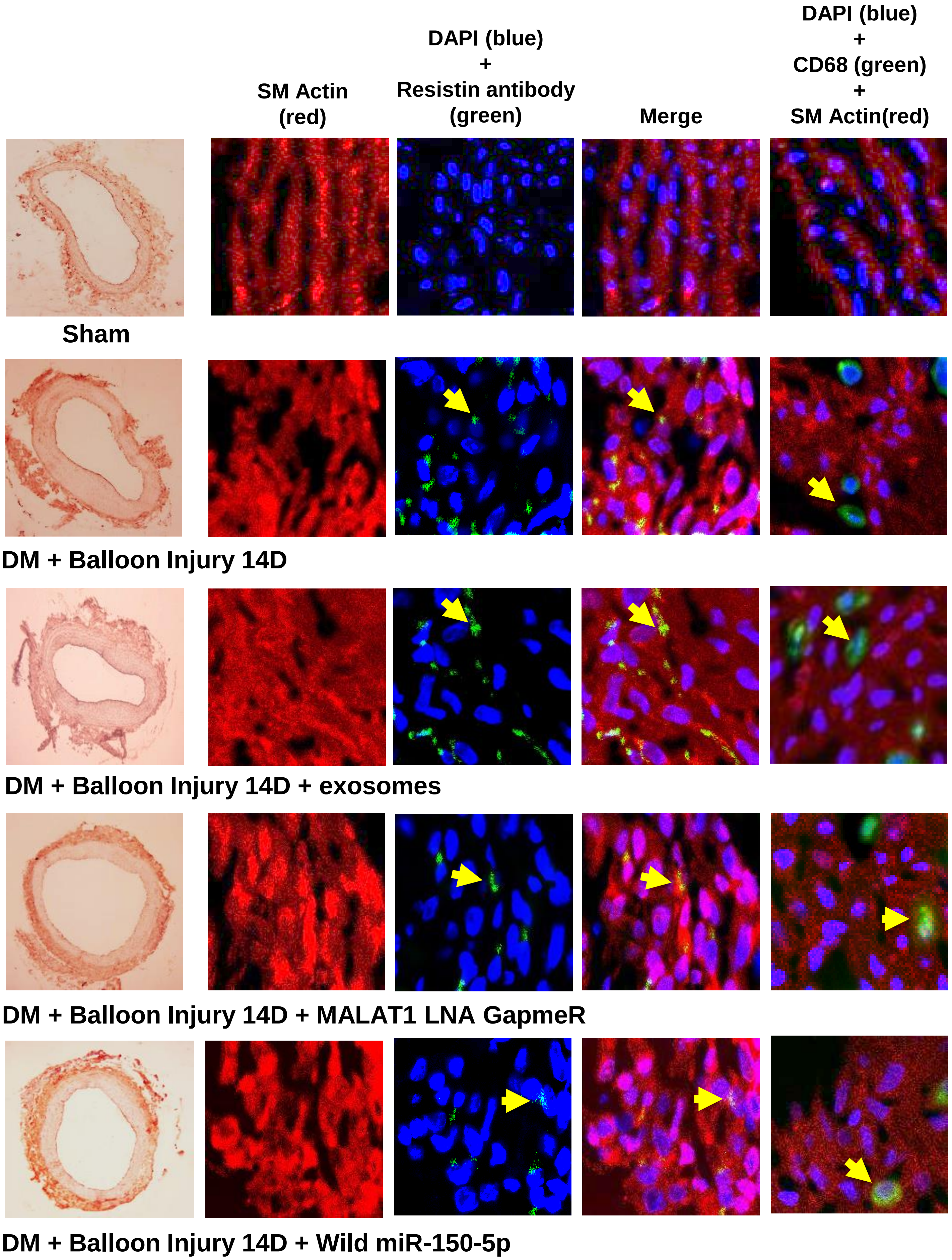

Supplemental Fig.SIII

Supplemental Figure legends

Supplemental Figure SI: Cell viability of macrophages after exogenous addition of macrophage-derived exosomes with different concentrations. The macrophage-derived exosomes were extracted from macrophages after 25 mM glucose treatment for 1 hour. N=4 per group.

Supplemental Figure SII: Effect ofexogenous addition of macrophage-derived exosomes on glucose uptake in macrophages. The macrophage-derived exosomes were extracted from macrophages after 25 mM glucose treatment for 1 hour.

*P<0.01 vs. control. N=4 per group.

Supplemental Figure SIII: The macrophage-derived exosomes increases resistin protein labeling after carotid artery injury. Immunofluorescence staining of the intimal area was performed at 14 days after carotid artery injury. Labeling of resistin and CD68 increased after balloon injury and macrophage-derived exosomes therapy. Silencing of MALAT1 by MASLAT1 LNA GapmeR and overexpression of miR-150-5p decreased the labeling induced by carotid artery injury. The resistin positive labeling cells were VSMCs, which were proved by positive anti-SMC actin staining.

Supplemental Methods

Cell viability assay

Cell viability was measured using the CellTiter-Glo® luminescent cell viability assay kit (Promega Corporation, Madison, WI, USA) according to manufacturer's instructions. Briefly, macrophages were grown and assayed in DMEM culture medium at 25,000 cells per well in a 96-well plate. After an equal volume of CellTiter-Glo® reagent was added, plates were shaken and then incubated at room temperature for 10 min to stabilize the luminescence signal. Finally, cell viability was assessed as the relative luminescence unit by using a luminometer (Glomax, Promega Corporation, Madison, WI, USA).

Assessment of glucose uptake

Cells were plated at a density of 10,000 cells/well on a 96-well plate. The following day, cells were transfected with various vectors and serum-starved. During the next day, cells were either left untreated or stimulated with macrophage-derived exosomes 50μg (high glucose treated) in groups. After an additional 5-hour incubation, glucose uptake was measured using Glucose Uptake-Glo Assay (Promega) according to the manufacturer's instructions.

Histological and immunofluorescence staining

The tissue samples of carotid artery were harvested and treated overnight in 4% paraformaldehyde and then embedded in paraffin. The tissues were cut into 5-μm-thick sections and incubated with the primary antibody[anti-resistin antibody (CHEMICON, Damstadt, Germany),CD68 antibody (SANTA CRUZ BIOTHCHNOLOGY, INC. USA)or mouse monoclonal anti-SMC actin antibody (SANTA CRUZ BIOTHCHNOLOGY, INC. USA) at4°C for 12 hours. The sections were then washed thrice with PBS, incubated with fluorescence-conjugated secondary antibody in PBS for 1-2 hours at room temperature in the dark and then stained with DAPI to visualize the nuclei. The sections were mounted with a coverslip and examined using fluorescence microscopy. Images were taken from at least three random fields for each sample.
